# Genome assembly and annotation of two clinical *Naegleria fowleri* isolates from Costa Rica

**DOI:** 10.1128/mra.00019-26

**Published:** 2026-09-03

**Authors:** Nina Allouch, Lissette Retana Moreira, Nalhyte Aurago, Elizabeth Abrahams Sandí, Isabel Marcelino

**Affiliations:** 1 Institut Pasteur de la Guadeloupe, Les Abymes, Guadeloupe, France; 2 Departamento de Parasitología, Facultad de Microbiología, Universidad de Costa Rica 27915, San José, Costa Rica; 3 Laboratorio de Investigación en Amebas de Vida Libre, Centro de Investigación en Enfermedades Tropicales (CIET), Universidad de Costa Rica 27915, San José, Costa Rica; University of California Riverside, Riverside, California, USA

**Keywords:** *Naegleria fowleri*, primary amebic meningoencephalitis, genome assembly, genome annotation

## Abstract

Here, we report the genome assembly and annotation of two clinical isolates of *Naegleria fowleri* from primary amebic meningoencephalitis cases in Costa Rica in 2020 using Illumina sequencing. Each assembly comprised 37 reference-guided scaffolds, with genome sizes of approximately 28 Mbp and a GC content of 36.87%.

## ANNOUNCEMENT

The free-living amoeba *Naegleria fowleri* (supergroup Excavata; phylum Percolozoa; class Heterolobosea) is distributed worldwide in freshwater and soil environments and causes primary amebic meningoencephalitis (PAM), which is highly fatal (>97%) (1). Although a genus-level *Naegleria* pangenome has provided important structural and functional insights into the diversity and adaptability of these free-living amoebae (2), the pathogenicity and biology of *N. fowleri* remain incompletely understood. Additional close-to-complete assemblies from clinical PAM cases, particularly from Central America, are needed to better represent pathogenic lineages. Here, we assembled and annotated two clinical isolates obtained in 2020 from PAM cases in the Guanacaste and Limón provinces of Costa Rica (3, 4).

*N. fowleri* Guanacaste (MT090627; NF_Guanacaste) and *N. fowleri* Limón (MT210902; NF_Limón) were isolated from cerebrospinal fluid and maintained axenically in 2% casein hydrolysate at 37°C (3, 4). Genomic DNA was extracted from approximately 1 × 10⁷ trophozoites using the QIAamp DNA Mini Kit with RNase A treatment, quantified with NanoDrop, and sequenced (2 × 150 bp) on a NextSeq 500 system with Nextera XT libraries at the Biomics Core Facility (Institut Pasteur, France). Reads were assessed with FastQC v0.12.1 (5), trimmed with Cutadapt v5.0 (6), and filtered with Trimmomatic v0.39 (7) (LEADING:5, TRAILING:5, SLIDINGWINDOW:4:20, and MINLEN:36). Default parameters were used except where otherwise noted.

*De novo* genome assembly was performed with SPAdes v4.0.0 (8) and evaluated with QUAST v5.3.0 (9) and BUSCO v5.7.1, using eukaryota_odb10 (255 markers) in genome mode (10). Contigs were scaffolded with RagTag v2.1.0 using the scaffold command against the *N. fowleri* TY reference genome (NF_TY) (11). Final coverage was estimated by mapping Illumina reads to each reference-guided assembly with BWA-MEM v0.7.18 and calculating mean depth with samtools. Repeats were modeled with RepeatModeler v2.0.5 and masked with RepeatMasker v4.1.6 using the Dfam database before MAKER v2.31.11 gene prediction, which followed previously described procedures (2). Protein-coding genes were predicted using evidence-based alignments, the *ab initio* predictors SNAP and AUGUSTUS, genome-guided transcript assemblies from public *N. fowleri* RNA-seq data (ENA project PRJNA642022; NF1_LV and NF45_HV strains from Guadeloupe) (12), and Swiss-Prot proteins as evidence.

Annotation completeness was assessed using BUSCO in transcriptome mode on MAKER-predicted transcripts and using Annotation Edit Distance (AED). AED scores were calculated using the AED_cdf_generator.pl script (13); gene models with AED ≤0.5 represented 98.0% and 97.8% of NF_Guanacaste and NF_Limón, respectively. Shared and strain-specific genes were identified using MAKER-predicted protein sequences with the MMseqs2 easy-rbh workflow with default parameters (14, 15).

Final reference-guided draft assemblies comprised 37 scaffolds, with genome sizes of 27.95 and 27.97 Mbp, guanine-cytosine (GC) contents of 36.87%, and mean sequencing depths of 341× and 270× for NF_Guanacaste and NF_Limón, respectively. Scaffold ends were not determined to be complete. Gene prediction identified 9,082 and 8,920 protein-coding genes in NF_Guanacaste and NF_Limón, respectively. BUSCO completeness was nearly 88% in genome mode and nearly 80% in annotation transcriptome mode (Table 1). Comparative analysis identified 8,306 shared genes, with 714 genes unique to NF_Guanacaste and 542 unique to NF_Limón.

**TABLE 1:** Genome sequencing, assembly, and annotation statistics for *N. fowleri* isolates NF_Guanacaste and NF_Limón

Genome features	NF_Guanacaste	NF_Limón
Raw reads	59,622,891	37,983,163
Q30 reads (%)	89.24	88.69
Contigs number (SPAdes)	6,756	4,191
N50 (bp) (SPAdes)	79,683	101,789
Final reference-guided scaffolds	37	37
Final assembly size (Mbp)	27.95	27.97
GC content (%)	36.87	36.87
Reads mapping to SPAdes assemblies (%)	94.69	97.00
Mean sequencing depth, final assembly	341×	270×
Number of protein-coding genes	9,082	8,920
BUSCO in final assembly, genome mode, eukaryota_odb10 (Complete, Fragmented, Missing)	C: 87.1%, F: 2.7%, M: 10.2%	C: 88.3%, F: 2.4%, M: 9.3%
BUSCO in annotation, transcriptome mode, eukaryota_odb10 (Complete, Fragmented, Missing)	C: 79.2%, F: 7.8%, M: 13.0%	C: 80.4%, F: 7.8%, M: 11.8%
Gene models with AED ≤0.5	98.0%	97.8%

## Data Availability

Raw reads for *Naegleria fowleri* strains NF_Guanacaste and NF_Limón are available in the NCBI Sequence Read Archive under BioProject PRJNA1397228, with accession numbers SRR36661213 and SRR36661214. The Whole Genome Shotgun assemblies and corresponding MAKER annotations have been deposited in DDBJ/ENA/GenBank under accession numbers JBXTWM000000000 and JBXTWL000000000. The versions described in this paper are JBXTWM010000000 and JBXTWL010000000.
